# A "Matron's Corner"

**Published:** 1887-09-03

**Authors:** 


### A "MATRON'S CORNER."

Please send me The Hospital for the next six weeks and complete the quarter, afterwards I will send stamps for next quarter. I don't see your charges for advertisements anywhere. Have you a scale? Don't you think a "Matron's Corner" would be useful, where we might hold friendly discussions on other than purely nursing subjects? When we meet we (I speak from a long experience) have many points of interest to discuss.

August 24, 1887. Matron.

*** We shall be glad to know what other matrons have to say of this suggestion about a "Matron's Corner." The scale of charges for advertisements is published each week at the head of the first *Notes and News* column.—[Ed. T. H.]

